# Sexual Selection on male cuticular hydrocarbons via male–male competition and female choice

**DOI:** 10.1111/jeb.12875

**Published:** 2016-04-27

**Authors:** S. M. Lane, A. W. Dickinson, T. Tregenza, C. M. House

**Affiliations:** ^1^ Centre for Ecology and ConservationCollege of Life and Environmental SciencesUniversity of ExeterCornwallUK

**Keywords:** antagonistic selection, cuticular hydrocarbons, female mate choice, *Gnatocerus cornutus*, male–male competition, sexual conflict, sexual selection

## Abstract

Traditional views of sexual selection assumed that male–male competition and female mate choice work in harmony, selecting upon the same traits in the same direction. However, we now know that this is not always the case and that these two mechanisms often impose conflicting selection on male sexual traits. Cuticular hydrocarbons (CHCs) have been shown to be linked to both social dominance and male attractiveness in several insect species. However, although several studies have estimated the strength and form of sexual selection imposed on male CHCs by female mate choice, none have established whether these chemical traits are also subject to sexual selection via male–male competition. Using a multivariate selection analysis, we estimate and compare sexual selection exerted by male–male competition and female mate choice on male CHC composition in the broad‐horned flour beetle *Gnatocerus cornutus*. We show that male–male competition exerts strong linear selection on both overall CHC abundance and body size in males, while female mate choice exerts a mixture of linear and nonlinear selection, targeting not just the overall amount of CHCs expressed but the relative abundance of specific hydrocarbons as well. We discuss the potential implications of this antagonistic selection with regard to male reproductive success.

## Introduction

The widespread elaboration of male sexual traits results from two mechanisms of sexual selection first proposed by Darwin – male–male competition and female mate choice. A long‐held assumption of sexual selection was that these two mechanisms should be reinforcing (i.e. impose the same form and direction of selection on the same suite of male traits), resulting in a scenario whereby females always preferred dominant males (Cox & Le Boeuf, 1977; Berglund *et al*., 1996; Wiley & Poston, 1996). However, a large body of evidence now demonstrates that the selection pressures of male–male competition and female mate choice are often conflicting (reviewed in Qvarnström & Forsgren, 1998; Wong & Candolin, 2005).

Nevertheless even when mechanisms of sexual selection are antagonistic and females do not exert a preference for dominant males, it remains possible for dominant males to gain a mating advantage over their competitors through force or coercion, increasing their own mating opportunities and ultimately overriding female mate choice (Qvarnström & Forsgren, 1998; Wong & Candolin, 2005). Evidence of conflict between male–male competition and female mate choice has been observed in flour beetles (Harano *et al*., 2010; Yamane *et al*., 2010; Okada *et al*., 2014), cockroaches (Moore & Moore, 1999), bitterlings (Reichard *et al*., 2005; Casalini *et al*., 2009), brown trout (Petersson *et al*., 1999) and water striders (Sih *et al*., 2002). The consequences of mating with dominant, nonpreferred males can be severe, for example female cockroaches *Nauphoeta cinerea* mated to nonpreferred males had a reduced lifespan and produced fewer offspring (Moore *et al*., 2001, 2003). This effect is linked to dominant male's pheromone composition – a sexually selected trait in this species (Moore *et al*., 2003).

Although chemical cues have received relatively little attention in comparison with more conspicuous male sexual traits (e.g. visual and acoustic traits), recent years have seen a surge in studies investigating the role of chemical traits in determining male mating success (Wyatt, 2014). Chemical cues have now been shown to signal a multitude of male characteristics including dominance status (e.g. cockroaches – Moore *et al*., 1997; South *et al*., 2011; field crickets – Thomas & Simmons, 2009a), condition (e.g. meadow voles – Ferkin *et al*., 1997; Hobbs & Ferkin, 2011), infection status (reviewed in Penn & Potts, 1998) and even genetic compatibility (e.g. field crickets – Thomas & Simmons, 2011a; Capodeanu‐Nägler *et al*., 2014). Cuticular hydrocarbons (CHCs), semiochemicals found on the cuticles of most terrestrial arthropods, have been shown to be particularly important in providing cues of a male's socio‐sexual environment and signals of male quality [see Ingleby, 2015 for a review] (as well as playing a key role in species and mate recognition [see Howard & Blomquist, 2005 & Johansson & Jones, 2007 for a review]) in insects.

Cuticular hydrocarbons are known to convey information about male competitive ability and attractiveness. For example, male CHC profiles often determine the outcome of both male–male competition (e.g. cockroaches – Roux *et al*., 2002; field crickets – Kortet & Hedrick, 2005; Thomas & Simmons, 2009a, 2011b) and female mate choice (e.g. field crickets – Kortet & Hedrick, 2005; Ivy *et al*., 2005; Thomas & Simmons, 2009b; Simmons *et al*., 2013; Steiger *et al*., 2013, 2015; *Drosophila* – Blows, 2002; Howard *et al*., 2003; Chenoweth & Blows, 2005; Ingleby *et al*., 2014). However to date, only a handful of studies, limited to *Drosophila* and field crickets, have investigated the strength and form of sexual selection imposed on CHCs (reviewed in Steiger & Stökl, 2014). Furthermore, these few studies only look at the strength and form of sexual selection imposed by female mate choice not by male–male competition. As discussed above, sexual selection during one episode of selection may not be reflective of selection occurring at another. Therefore, it cannot be assumed that selection on CHCs during male–male competition is reinforced during selection on CHCs from female mate choice (Hunt *et al*., 2009).

Here, we examine the form and strength of sexual selection imposed by male–male competition and female mate choice on male CHCs in the broad‐horned flour beetle *Gnatocerus cornutus*. Male–male competition is an important component of the mating system of *G. cornutus*. Males possess enlarged mandibles, a trait absent in females, which they use to fight over territories and mates. Mandible size is a major determinant of fight outcome, and although females do not prefer males with large mandibles or males that win fights, evidence suggests that aggressive males are still able to secure a mating advantage under competitive scenarios (Harano *et al*., 2010; Yamane *et al*., 2010; Okada *et al*., 2014). Cuticular hydrocarbon profiles are sexually dimorphic in *G. cornutus*, consisting of 24 compounds (previously identified using gas chromatography) which vary in concentration between the sexes (Lane *et al*., 2015). Males of this species are able to detect slight changes in female CHC profiles brought about by previous contact with males and respond to the risk and intensity of sperm competition that these chemical cues signal (Lane *et al*., 2015), indicating that CHCs are an important source of information.

We begin by using a multivariate approach to estimate the strength of linear and nonlinear sexual selection on male CHCs during both male–male competition and female mate choice. We then test whether the strength and form of sexual selection on male CHCs changes across these two contexts of selection. As previous studies have demonstrated that male–male competition and female choice do not favour the same traits in *G. cornutus*, we predict that sexual selection on male CHCs will be significantly different under the two contexts of sexual selection.

## Materials and methods

### Stock populations and rearing protocols

Beetles used in this study were taken from stock populations of *G. cornutus* derived from the Japanese National Food Research Institute (NFRI), at which beetle cultures have been maintained for over 50 years (see Okada *et al*., 2006 for details of origin and culture conditions). In our laboratory, mixed sex populations have been maintained since 2012 in pots (Thermoscientific Nalgene 500 mL, 120 mm OD) containing 100 individuals (50 ♀ and 50 ♂). These stock populations are reared on wholemeal wheat flour enriched with 5% yeast and incubated at 27 °C and 60% humidity on a 14L: 10D lighting cycle (Okada *et al*., 2006). Every 3–4 weeks, final‐instar larvae are randomly removed from each stock pot (*n* = 18 pots) and placed into six 24‐well plates as pupation is inhibited at moderate‐to‐high larval density (Tsuda & Yoshida, 1985). At eclosion, 50 male and 50 female adults are randomly selected to form the parents of the next generation of each pot.

For the purposes of this study, final‐instar larvae were collected from laboratory stocks daily and placed into 24‐well plates until eclosion. After eclosion, adults were separated by sex, transferred into fresh 24‐well plates and provided with *ad libitum* wholemeal wheat flour. To ensure sexual maturity, behavioural trials were run 7–15 days after eclosion.

### Fighting success trials

Twenty‐four hours before fighting trials, males were randomly allocated as either the focal or nonfocal male. Those allocated as nonfocal males were marked with white correction fluid on their elytra and returned to individual cells to allow the correction fluid to dry. Focal males would later be used for CHC analysis and therefore could not be marked. Marking has no effect on the outcome of male–male competition (*CMH personal observation*). The next day, focal and nonfocal males were randomly paired in fighting arenas and observed for 20 min. All acts of aggression were recorded along with the winner of each bout. Aggressive encounters were classified as: (1) Males repeatedly placed their mandibles beneath an opponent's body and attempted to lift and flip them onto their back. (2) Males shoved and bit their opponent with their mandibles (Okada *et al*., 2006). Losers were determined as those who were successfully flipped onto their back or who retreated from the encounter under the pursuit of the victor. At the end of the 20 min, focal individuals were frozen at −20 °c for subsequent chemical analysis. For each focal individual, the number of wins and losses was tallied to reveal whether they won or lost the majority of aggressive encounters. Each focal individual was then assigned an overall fight score of either 1 (*n* = 283) or 0 (*n* = 217) for winning or losing, respectively.

### Mating success trials

Males and females were paired randomly in mating arenas and observed for 20 min, during which time we noted all courtship attempts and successful copulations. Males have a stereotypical courtship display which commences when a male mounts a female and drums her abdomen with his tibia. This may be followed by a brief copulation that lasts only 3–4 s. Males who obtained a mating were given a fitness score of 1 (*n* = 353), while those who exhibited courtship behaviour but failed to obtain a mating were given a fitness score of 0 (*n* = 147). All males were removed from the mating arena at the completion of the trial and were frozen at −20 °C for CHC extraction.

### CHC extraction and analysis

Samples were randomized before the extraction process to eliminate any possible effects of column degradation over time. Cuticular hydrocarbons were then extracted from individuals via full‐body immersion in 50 *μ*L of HPLC‐grade hexane with 10 ppm pentadecane as an internal standard. Individual beetles were left to soak for 5 min, during the final minute of which they were vortexed to maximize CHC extraction. After the 5 min, beetles were removed from the extraction vials using metal forceps which were cleaned in pure hexane between samples to avoid contamination. Individual beetles were then placed back into their corresponding Eppendorf tubes to be re‐frozen for later body measurements.

A total of 2 *μ*L of the extracted CHC sample was injected into a GC‐FID (gas chromatograph with flame ionization detector – Agilent 7890) fitted with two injectors, and two DB‐1 columns (of 30 m × 0.25 mm with an internal diameter of × 0.25 *μ*m film thickness) using helium as a carrier gas. The inlets were set at 250 °C, and the injection was run in the pulsed splitless mode. The GC oven temperature profile started at 100 °C for 1 min, ramping at 20 °C min^−1^ to 250 °C, then finally 5 °C min^−1^ to 320 °C. The FID detector heaters were set at 300 °C, the H_2_ flow was 22 mL min^−1^, and the air flow was 200 mL min^−1^. Nitrogen was used to make up the column flow to 30 mL min^−1^.

### Morphological measurements

We captured images of the dorsal view of the focal males' bodies using a Leica M125 microscope with mounted camera (Leica DFC295, Leica microsystems Ltd. CH‐9435 Heerbrugg) which conveyed images to a PC. We then measured the width of the pronotum (to the nearest 0.01 mm) as an index of body size (Okada *et al*., 2006) using ImageJ (version 1.46r, National Institutes of Health, Bethesda, MD, USA). We measured a subset of these pronota twice to calculate the repeatability of this measure (using the R code of Wolak *et al*., 2012 and the R package ‘ICC’) and found that the repeatability is high (Pronotum width = 0.98, 95% CIs: 0.99, 0.96 *P *<* *0.001).

### Statistical analyses

#### Principal components analysis

The GC‐FID analysis identified 24 individual CHC peaks. After dividing all peaks by the internal standard (peak 1), the resulting data were log_10_ transformed. We pooled the data from both of our data sets (i.e. male–male competition and female mate choice) and ran a principal components analysis (PCA) to obtain a reduced number of eigenvectors which capture and describe the variation in CHC profiles. All data analysis was carried out using SPSS (version 20, IBM Corp, Armonk, NY, USA).

#### Measuring sexual selection

##### Multivariate selection analysis

Individuals in our analysis are independent as males were used once only (i.e. for either mating or fighting trials). We quantified the strength and form of sexual selection acting on male CHCs across the two contexts of selection using a standard multivariate selection analysis (Lande & Arnold, 1983). Before running the selection analyses, we first calculated individual relative fitness by dividing individual absolute fitness scores by the mean fitness score for each population of males within each selection context [e.g. a male with an absolute fitness score of 1 would have a relative fitness score of 1/(pop mean fitness)] (Hunt *et al*., 2009). Converting absolute fitness into relative fitness makes our selection gradients directly comparable to those of other studies, facilitating comparative studies. Furthermore, because our explanatory variables (pronotum width and CHC concentration) were measured in different units, it was necessary to standardize them to have a mean of zero and a standard deviation of one for statistical comparison. Our CHC data were already standardized by the principal components analysis conducted on our full data set (see section above) while pronotum width (PW) was standardized using a Z‐transformation.

We then fitted linear regression models separately for each context of selection to obtain estimates of standardized linear selection gradients acting on male CHC composition and body size during male–male competition (***β***_**mc**_) and mating success (***β***_**ms**_). These linear regression models included standardized PW and the 3 PCs obtained from our PCA as the predictor variables with relative fitness as the response variable. Next, we calculated the quadratic and correlational terms by multiplying the explanatory/predictor variables either by themselves (quadratic) or by each other (correlational). Using these terms, we then fitted a quadratic regression model (again separately for each selection context) incorporating all linear, quadratic and correlational terms to estimate the matrix of nonlinear selection gradients during male–male competition (***γ***_**mc**_) and mating success (***γ***_**ms**_). Regression models underestimate quadratic selection by a factor of 0.5, so we doubled the quadratic selection gradients as recommended by Stinchcombe *et al*. (2008). Furthermore, as our measures of fitness (fighting and mating success) did not conform to a normal distribution, we assessed the significance of our linear and nonlinear selection gradients for each data set using a resampling procedure where fitness scores were randomly shuffled across individuals in the data set to obtain a null distribution for each gradient where there is no relationship between trait and fitness (Mitchell‐Olds & Shaw, 1987). Probabilities are the number of times (out of 9999 permutations) in which the gradient pseudo‐estimate was equal to or less that the original estimated gradient. We conducted separate randomization analyses for the multiple regression models for directional selection (i.e. model containing only linear terms) and for the full quadratic model (i.e. model containing linear, quadratic and correlational terms).

As the strength of nonlinear selection can be underestimated by individually interpreting the size and significance of *γ*‐coefficients (Phillips & Arnold, 1989; Blows & Brooks, 2003), we conducted a canonical analysis to locate the major axes of nonlinear selection described by ***γ***_**mc**_ and ***γ***_**ms**_ (Phillips & Arnold, 1989; Reynolds *et al*., 2010) using the car package of R (see Supporting Information, Appendix S1 for the R script). Permutation tests of the eigenvalues were conducted by randomly shuffling the fitness variable 10 000 times for each simulated data set to estimate the numbers of times the observed F statistic exceeded the F statistic from the permuted data sets (Reynolds *et al*., 2010). This tests whether the test statistic of the eigenvectors extracted from the canonical analysis of ***γ***_**mc**_ and ***γ***_**ms**_ is larger than expected by chance (Reynolds *et al*., 2010).

##### Visualizing the fitness surface

To visualize the fitness surfaces from the canonical rotation of ***γ***_**mc**_ and ***γ***_**ms**_, we used thin‐plate splines (Green & Silverman, 1994). We used the Tps function in the field package of R (version 2.13.0; available via http://www.r-project.org) to fit spline surfaces using the value of the smoothing parameter (*λ*) that minimized the generalized cross‐validation (GCV) score. We then plotted perspective surfaces and contour maps in R.

Finally, we used a sequential model building approach (as outlined in Appendix A of Chenoweth & Blows, 2005) to examine whether the form and strength of sexual selection acting on male CHCs differed significantly between the two contexts of selection (male–male competition and female choice). In short, this approach tests for differences in the sign and curvature of the linear, quadratic and correlational selection gradients across the two selection contexts. It does so by comparing the variance explained by a regression model that fits a single relationship through the two selection contexts with a regression model that fits a separate relationship for each selection context. If the second model explains significantly more variance than the first, as determined by a partial F test, this demonstrates that selection gradients differ significantly across the two selection contexts. For a detailed outline of this approach, see Appendix S2.

## Results

### Principal components analysis

GC‐MS analysis identified 24 individual CHC peaks. Individual profiles consisted of a mixture of straight‐chained alkanes, mono‐ and dimethyl alkanes, and alkenes ranging in length from 25 to 33 hydrocarbons (see Supporting Information for more details). Principal components analysis resulted in three significant PCs (with eigenvalues of > 1) that collectively explained 80.6% of the total variation in male CHC expression (see Table 1) and CHCs with factor loadings ≥0.25 were considered to have contributed significantly to that PC (Tabachnick & Fidell, 1989). PC1 explained 60.4% of the variance and was heavily positively loaded to all CHC peaks except for peak 16 to which it was negatively loaded; thus, we interpreted PC1 as representing overall investment in CHC production. PC2 explained another 14.3% of the variance and was significantly loaded to 16 of 24 CHCs, some positively and some negatively. PC3 explains a further 5.8% of the variance and was only significantly loaded to 5 CHCS. Due to the complex nature of these loadings, PC2 and PC3 were interpreted as representing specific blends of CHCs that are present in larger or smaller amounts. For example, PC3 was positively loaded to peaks 1, 3, 11 and 16 and negatively loaded to peak 17 so represents a trade‐off between four different peaks and a single peak.

**Table 1:** Results of principal components analysis for male CHCs. Compounds with a loading factor > 0.25 were classified as biologically significant and are shown in bold (Tabachnick & Fidell, 1989). CHCs are listed in ascending order of chain length

		PC1	PC2	PC3
	Eigenvalue	14.501	3.442	1.394
	% variance	60.423	14.341	5.808

Peak no.	*Loadings*			
	Compound ID			
1	C_25_	**0.541**	−0.062	**0.732**
2	11‐MeC_25_	**0.797**	**0.396**	−0.096
3	3‐MeC_25_	**0.758**	**0.385**	**0.408**
4	C_26_	**0.882**	−**0.261**	0.230
5	11‐MeC_26_	**0.825**	**0.439**	−0.091
6	5‐C_26_‐ene	**0.458**	0.246	−0.176
7	C_27_	**0.739**	−**0.387**	0.240
8	11‐MeC_27_	**0.869**	**0.358**	0.149
9	Unknown	**0.792**	0.056	−0.249
10	11,15‐diMeC_27_	**0.654**	**0.355**	−0.172
11	3‐MeC_27_	**0.911**	−0.019	**0.257**
12	C_28_	**0.862**	−**0.378**	−0.006
13	C_29_	**0.738**	−**0.594**	0.079
14	13‐MeC_29_	**0.890**	0.201	0.019
15	11,15‐diMeC_29_	**0.772**	−**0.568**	−0.030
16	3‐MeC_29_	−**0.711**	**0.537**	**0.323**
17	C_30_	**0.723**	0.246	−**0.302**
18	C_31_	**0.669**	−**0.661**	−0.153
19	15‐MeC_31_	**0.893**	**0.288**	−0.067
20	3,19,3,17‐diMeC_31_	**0.771**	**0.389**	−0.221
21	3‐MeC_31_	**0.675**	−**0.630**	−0.186
22	4,12‐diMeC_31_	**0.828**	0.013	−0.102
23	11‐MeC_33_	**0.880**	0.216	0.137
24	15,17‐diMeC_33_	**0.827**	**0.284**	−0.101

### Male fighting success

**Figure 1:**
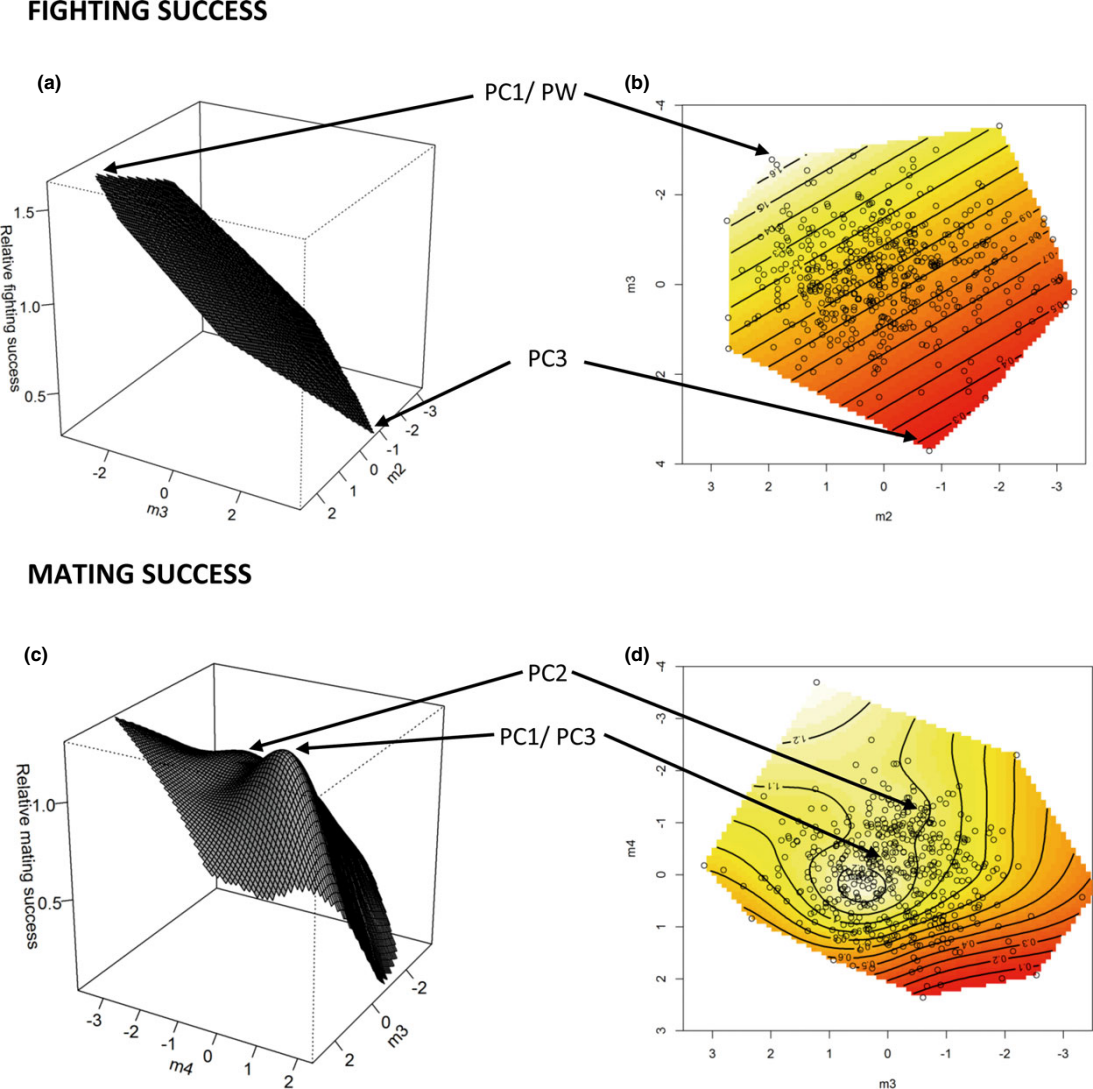
Thin‐plate spline visualization of the major canonical axes for fighting success (a and b) and mating success (c and d). The three‐dimensional surfaces on the left (a and c) show a perspective view while the contour plots on the right (b and d) show the same surfaces from above. The highest peaks are labelled with the traits that contribute most strongly to these regions of high/low fitness when the coefficients of both eigenvectors are interpreted together. Points on the contour plots represent actual males and white/very pale yellow areas indicate areas of highest/high fitness whereas red areas indicate areas of lower fitness.

Our selection analysis revealed significant negative linear selection acting on PC3 and positive linear selection acting on pronotum width (Table 2). Our analysis did not reveal any significant nonlinear gradients of selection being imposed by male–male competition. Canonical analysis of the *γ* matrix showed significant directional selection on two negative eigenvectors (m3 & m4) and two positive eigenvectors (m1 & m2) (Table 3). Visualization of the fitness surface along the most statistically significant axes (m2 & m3) indicated that overall, males who achieved the highest fighting success possessed both a larger body size (PW) and a greater overall CHC abundance (as represented by PC1). Low fighting success on the other hand was associated with high levels of alkane C25 and the three 3‐methylalkanes (peaks 1, 3, 11 and 16) represented by PC3 (Fig. 1a,b).

**Table 2:** The vector of standardized directional selection gradients (***β***), and the matrix of quadratic and correlational selection gradients (***γ***) for male CHC expression (i.e. PCs) and body size (PW) with respect to fighting and mating success in *G. cornutus*

	***β***	***γ***			
		PC1	PC2	PC3	PW
Fighting success					
PC1	0.056	0.018			
PC2	0.024	0.050	−0.024		
PC3	−**0.158** ***	0.057	0.020	0.006	
Pronotum width (PW)	**0.171** ***	0.025	−0.073	−0.040	−0.088
Mating success					
PC1	−**0.152** ***	−**0.138** **			
PC2	−0.002	0.062	−0.082		
PC3	−**0.151** ***	−0.034	0.007	−**0.112** **	
Pronotum width (PW)	**0.063** *	0.037	0.002	−0.029	−0.018

*<0.05, **<0.01, ***<0.001 Significant values (*P *<0.05 after randomization tests) are shown in bold.

**Table 3:** The **M** matrix of eigenvectors from the canonical analysis of ***γ*** for male CHC (i.e. PCs) and body size. ***θ***_***i***_ is the strength of directional selection and ***λ***_***i***_ is the strength of nonlinear selection along each of the eigenvectors m1–m4 across the two contexts of sexual selection

	**M**				Selection	
	PC1	PC2	PC3	PW	***θ***_***i***_	***λ***_***i***_
Fighting success						
m1	−0.625	−0.477	−0.575	0.225	**0.083** *	0.099
m2	0.607	−0.523	0.008	0.598	**0.122** ***	−0.001
m3	−0.367	−0.497	0.783	−0.073	−**0.169** ***	−0.031
m4	0.326	−0.501	−0.237	−0.766	−**0.087** *	−0.159
Mating success						
m1	−0.384	−0.254	0.291	−0.840	−0.038	0.011
m2	0.401	0.821	0.065	−0.410	−**0.098** ***	−0.053
m3	0.210	−0.203	−0.893	−0.343	**0.082** **	−**0.115** ***
m4	0.806	−0.473	0.341	−0.110	−**0.179** ***	−**0.194** *

*<0.05, **<0.01, ***<0.001 Significant values (*P *<* *0.05 after randomization tests) are indicated in bold.

### Male mating success

Female choice imposed significant negative linear selection on PC1 and PC3 but positive linear selection on pronotum width. Our selection analysis also revealed significant stabilizing (negative *γ*) selection acting on both PC1 and PC3 (Table 2). Our canonical analysis produced two major axes of nonlinear selection (m3 & m4). Visualization of the fitness surface against these two major axes indicated that in contrast to fighting success, mating success was highest for males who possessed low‐to‐intermediate overall CHC abundance (represented by PC1) alongside low levels of the C25 alkane and three 3‐methylalkanes represented by PC3 (Fig. 1c,d).

### Comparison of sexual selection across male–male competition and female mate choice

There was no significant difference in gradients of linear selection (*F*_4,990_ = 1.754, *P *=* *0.136) or correlational selection (*F*_6,970_ = 0.864, *P *=* *0.521) acting on male CHCs and body size between the two contexts of selection. However, quadratic selection differed significantly between the two contexts of sexual selection (*F*_1,970_ = 4.268, *P *=* *0.039). This difference in quadratic selection was a consequence of stabilizing selection acting on the overall (i.e. PC1) and relative abundance of specific CHCs (i.e. PC3) as a result of female mate choice, while male–male competition did not exert any quadratic selection on CHCs at all.

## Discussion

Male CHCs are known to be associated with social dominance and fight outcomes (Roux *et al*., 2002; Kortet & Hedrick, 2005; Thomas & Simmons, 2009a, 2011a), but to date, to our knowledge, no one has measured the form of sexual selection imposed on male CHCs by male–male competition. Here, by measuring sexual selection exerted by male–male competition and female mate choice, we show that male CHCs in *G. cornutus* are subject to strong sexual selection via both mechanisms, and furthermore that the strength and form of selection exerted on male CHCs differ significantly between the two.

Male–male competition exerted strong linear sexual selection on male CHC composition and body size. Overall, CHC abundance (PC1) and body size were subject to positive linear selection, indicating that large males with a high overall amount of CHCs had the greatest fighting success. Males who were least successful in fights had high levels of specific CHC blends (PC3), suggesting that fighting success is maximized by increased investment in overall CHC abundance and decreased investment in relative amounts of specific CHCs. Female choice on the other hand imposed linear and stabilizing nonlinear sexual selection on both the overall abundance of CHCs and on specific CHC blends. For instance, mating success was highest for males with a low‐to‐intermediate total amount of CHCs and low‐to‐intermediate levels of the specific CHC blend represented by PC3. In this study, PC3 was heavily loaded to 3‐methylalkanes and was under negative sexual selection by both male–male competition and female choice. Previous studies have found strong evidence that 3‐methylalkanes are associated with fertility and immune status in female social insects (Holman, 2012; Holman *et al*., 2010; Oystaeyen *et al*., 2014) and are thus thought to signal high quality. The pattern is reversed in our study, high levels of 3‐methylalkanes were associated with both low fighting success and low mating success in males, indicating that these compounds do not represent high quality in *G. cornutus*. Although sexual selection on male CHCs was statistically significant in two different contexts, we cannot rule out the possibility that male CHCs are correlated with another trait that influences fighting and/or mating success and thus are under indirect rather than direct selection. Nonetheless, the patterns that we found are consistent with previous studies in crickets (Thomas & Simmons, 2009b; Simmons *et al*., 2013; Steiger *et al*., 2013, 2015) and *Drosophila* (Ingleby *et al*., 2014) which have shown similarly complex patterns of linear and nonlinear sexual selection acting on male CHCs as a result of female mate choice. More generally, complex sexual selection on multivariate signalling traits via female choice appears to be a common phenomenon. For example, females have been shown to impose similarly complex patterns of selection on acoustic signals such as male courtship song in crickets (Bensten *et al*. 2006; Simmons *et al*., 2013) and visual signals such as the intricate coloration of male guppies (Blows *et al*. 2003). Here, it appears that complex selection on male CHCs may reflect the complexity of the information that CHCs convey. Furthermore, our findings highlight that although overall trait expression is important (e.g. total CHC abundance), females are selecting upon multiple components within these composite traits.

Linear and correlational selection on male CHCs did not differ significantly between the two contexts of selection, but quadratic selection was found to be significantly different. This significant difference was due to the fact that female mate choice imposed stabilizing nonlinear selection on male CHC composition, whereas selection imposed by male–male competition was only linear in form. Specifically, fighting success was more strongly linked to an increased investment in overall CHC profile rather than to specific CHC blends. Mating success on the other hand was tightly linked to both a lower investment in overall CHC expression and a lower relative abundance of specific CHC blends. In some species of *Drosophila* and cricket (Ingleby *et al*., 2014; Steiger *et al*., 2015), overall CHC abundance has been found to correlate with male body size, potentially explaining why males with increased fighting success were both larger and possessed higher overall CHC abundance. Intrasexual selection commonly exerts positive directional selection on male sexual traits (Jones *et al*., 2012). However, we did not find evidence of correlational selection in this study. The form of selection imposed by female mate choice is more varied across the literature, with some studies showing female choice to target the relative abundance of specific CHCs as opposed to their overall abundance (Howard *et al*., 2003; Thomas & Simmons, 2009b; Simmons *et al*., 2013; Steiger *et al*., 2015) while other studies show as we have here that sexual selection targets both specific CHC blends as well as the total abundance of CHCs (Steiger *et al*., 2013; Ingleby *et al*., 2014).

Our findings indicate that sexual selection by male–male competition and female choice target different components of male CHC profile, and as a result, male CHCs linked with fighting success and mating success are not the same. This suggests that males who win fights may not also be able to achieve high mating success under female choice. A similar conclusion was reached in a recent study investigating female preference for morphological traits known to be associated with fighting success in this species (Okada *et al*., 2014). The authors found that females chose mates not on the basis of fighting traits but rather on male courtship rate, and consequently, mating success was not correlated with fighting success. Thus, like the aforementioned study, our results suggest that winning a fight does not equip males with any benefit when it comes to mating. However, antagonistic selection during male–male competition and female choice may not always restrict the mating success of dominant or aggressive males, namely if they are able to override this conflict. For instance in *G. cornutus*, males who win fights gain priority access to females and achieve a mating advantage under competitive scenarios (Harano *et al*., 2010; Yamane *et al*., 2010; Okada *et al*., 2014), circumventing female choice. Thus, although our study provides evidence that male–male competition and female choice are antagonistic, the consequences for males under competitive scenarios are more difficult to predict.

Although the findings of our study indicate a strong relationship between fighting success, mating success and male CHC expression, as with any correlational analysis, our results do not demonstrate that this relationship is causative. To verify that the sexual selection gradients in our study are indeed a result of direct selection on male CHCs and not some unmeasured yet correlated trait(s), it will be necessary to experimentally manipulate male CHCs and observe the resulting effect on male mating and fighting success. Although many studies have provided correlative evidence to suggest that CHCs are subject to strong sexual selection (reviewed in Steiger & Stökl, 2014), to date no one has used experimental manipulation to verify the effect of CHCs on fitness. Furthermore, although once thought to be fixed, evidence is increasingly demonstrating that CHC expression is a highly plastic trait (reviewed in Ingleby, 2015) and furthermore that both mating (Everaerts *et al*., 2010; Weddle *et al*., 2012) and fighting (Thomas & Simmons, 2009a, 2011b) can induce such changes. In this study, we measured male CHC expression after fighting and mating, and as the experimental design of our current study did not allow us to determine whether male CHC expression post‐fight/mating matches their pre‐fight/mating expression, further investigation will be required to determine whether we have indeed measured selection on CHC profiles as presented to potential opponents/mates.

An overwhelming number of studies of sexual selection on male CHCs have focused on the influence of female mate choice for male CHC profiles. Here, we provide the first evidence that male CHC composition can have a profound influence on the outcome of male–male competition and that sexual selection on male CHCs during fighting is opposed by sexual selection on CHCs during mating. However, studies incorporating experimental manipulation will be needed to show that these chemical traits are indeed important determinants of mating and fighting success and this is an area of research that we aim to address.

## Supporting information


**Appendix S1** R code for canonical analysis taken from Reynolds *et al*., 2010.
**Appendix S2** Methods for sequential model building approach used to compare episodes of sexual selection – taken from Chenoweth & Blows, 2005.
**Appendix S3** CHC profile composition.
**Figure S1** A typical GC profile obtained from solvent extracts of the cuticle of male *Gnatocerus cornutus*.
**Table S1** Chemical characterization of male CHCs in *Gnatocerus cornutus*.
