# Supplementary material for: Sexual Selection on male cuticular hydrocarbons via male–male competition and female choice
Source: J Evol Biol. 2016 Apr 27;29(7):1346–55. doi: 10.1111/jeb.12875 (PMC4999037; doi:10.1111/jeb.12875)
Supplement: Supplementary file 1 — Appendix S1 R code for canonical analysis taken from Reynolds et al., 2010. Appendix S2 Methods for sequential model building approach used to compare episodes of sexual selection – taken from Chenoweth & Blows, 2005. Appendix S3 CHC profile composition. Figure S1 A typical GC profile obtained from solvent extracts of the cuticle of male Gnatocerus cornutus. Table S1 Chemical characterization of male CHCs in Gnatocerus cornutus. [file JEB-29-1346-s001.pdf]

<u>**Supplementary material**</u>

APPENDIX A

R code for canonical analysis taken from Reynolds *et al.* 2010: -

```
################################################################################
# R Script for Permutation Based Hypothesis Tests of the Eigenvalues from a
# Canonical Analysis of the Gamma Matrix of Quadratic and Correlational
# Selection Gradients
# Questions and bug reports to npajewski at ms.soph.uab.edu
# Updated: 9/04/2009
################################################################################
#                          PROGRAM NOTES                              #
################################################################################
# 1. R available from http://www.r-project.org/
# 2. This program uses the "car" package for R, therefore this package needs to
# be installed prior to using the script. To install, type the following at the
# command prompt upon opening R.
install.packages("car", dependencies=TRUE)
# and then select an appropriate mirror for download (say USA(MI)).
# 3. Users simply need edit the file paths and parameter settings within the
# user input section. The entire code can then simply be copied
# (CNTL-A then CTNL-C) and pasted into the R window
################################################################################
#                    DESCRIPTION OF INPUT FILE FORMATS                    #
################################################################################
# The script expects 2 files + an optional covariates file as input. The default
# is to have these files in comma delimited format (.csv), although appropriate
# changes for tab delimited data are documented below.
```

```
# 1. Fitness components file. Each fitness component is a column, rows denote
# samples. This file can have multiple components.
# 2. Traits file. Rows index individuals (samples), columns index traits.
# 3. Covariates file. Same format as traits file, although inclusion of
# covariates is optional
# Note that each file should contain a header row containing names for the
# appropriate fitness component, trait, or covariate.
##################################################################################
#                        BEGIN USER INPUT SECTION                        #
#          (Note the direction of slashes in specifying file paths)          #
##################################################################################
dset_path<-"ISgenitalia_x.csv" # Path to traits file; e.g.,"F:/05trts.csv"
pset_path<-"ISfitness.csv" # Path to fitness components file; e.g.,"F:/05fit.csv"
cov_flag<-0 # 0=No covariates, 1=Include covariates
cov_path<-"...." # Path to covariates file; e.g.,"F:/05cov.csv"
trait_col<-1 # column to use in fitness components file
std_traits<-0 # Standardize traits? 1=Yes, 0=No
std_fitness<-0 # Convert fitness component to relative fitness, 1=Yes, 0=No
num_perm<-10000 # Number of permutations
piter<-100 # Print out progress from permutation testing every X permutations
######################## Reading in Datafiles ###################################
# Code is expecting comma delimited data, for tab delimited change to sep=" "
dset_input<-read.table(dset_path, header=TRUE, sep=",")
pset_input<-read.table(pset_path, header=TRUE, sep=",")
if(cov_flag==1){
  cset_input<-read.table(cov_path, header=TRUE, sep=",")
}
```

```
####################################################################################
#                         END USER INPUT SECTION                          #
####################################################################################
library(car)
cat("Cleaning out observations with missing fitness, trait, or covariate measurements...\n")
if(cov_flag==0){ # No covariates
  tset_input<-cbind(dset_input, pset_input[,trait_col])
  tset<-na.omit(tset_input)
  num_traits<-ncol(dset_input)
  fit<-tset[,num_traits+1]
  end_idx<-num_traits+1
  traits<-tset[,-end_idx]
  num_obs<-nrow(traits)
}else{ # Include covariates
  tset_input<-cbind(dset_input, cset_input, pset_input[,trait_col])
  tset<-na.omit(tset_input)
  srt_idx<-ncol(dset_input)+1
  end_idx<-ncol(dset_input)+ncol(cset_input)
  traits<-tset[,1:ncol(dset_input)]
  cset<-tset[,srt_idx:end_idx]
  fit<-tset[,end_idx+1]
  num_traits<-ncol(traits)
  num_obs<-nrow(traits)
  num_cov<-ncol(cset)
}
#################################
# Standardize traits and fitness
```

```
################################
if(std_fitness==1){
  cat("Converting to relative fitness....\n")
  relfit<-fit/mean(fit)
}else{
  relfit<-fit
}
relfit<-as.data.frame(relfit)
names(relfit)<-c("relfit")
if(std_traits==0){
  strait<-traits
}else{
  cat("Standardizing traits....\n")
  strait<-matrix(rep(0.0, num_obs*num_traits), nrow=num_obs)
  for(j in 1:num_traits){
    for(i in 1:num_obs){
      strait[i,j]<-(traits[i,j]-mean(traits[,j]))/sd(traits[,j])
    }
  }
}
strait<-as.data.frame(strait)
names(strait)<-names(traits)
# Create cross-product and quadratic terms for response surface model
cat("Creating cross-product and quadratic model terms....\n")
for(i in 1:num_traits){
  for(j in i:num_traits){
    newterm<-strait[,i]*strait[,j]
```

```
    temp_names<-names(strait)
    strait<-cbind(strait,newterm)
    names(strait)<-c(temp_names,paste(names(strait)[i],names(strait)[j],sep="_"))
  }
}
##################################
# Build formula object
##################################
cat("Fitting response surface model....\n")
if(cov_flag==1){
  cat("Including covariates in model....\n")
  jstrait<-strait
  strait<-cbind(strait,cset)
}
main_effects = paste(names(strait),collapse="+")
form1<-as.formula(paste(names(relfit),"~",main_effects,sep=""))
dset2<-as.data.frame(cbind(relfit,strait))
rsmod<-lm(form1,dset2)
cat("Constructing gamma matrix and calculating canonical coefficients....\n")
gamma<-matrix(rep(0.0, num_traits*num_traits), nrow=num_traits)
num_terms<-ncol(strait)
index<-num_traits+2
for(i in 1:num_traits){
  for(j in i:num_traits){
    if(i==j){
      gamma[i,j]<-2.0*rsmod$coefficients[index]
      gamma[j,i]<-gamma[i,j]
```

```
      index<-index+1
    }else{
      gamma[i,j]<-rsmod$coefficients[index]
      gamma[j,i]<-gamma[i,j]
      index<-index+1
    }
  }
}
m<-eigen(gamma)$vectors
obs_m<-m
can_coef<-eigen(gamma)$values
y<-as.matrix(strait[,(1:num_traits)])%*%m
for(i in 1:num_traits){
  for(j in i:num_traits){
    newterm<-y[,i]*y[,j]
    y<-cbind(y,newterm)
  }
}
temp_names<-c("Z_1")
numc<-ncol(y)
for(i in 2:numc){
  temp_names<-c(temp_names,paste("Z",i,sep="_"))
}
Z<-as.data.frame(y)
names(Z)<-temp_names
if(cov_flag==1){
  Z<-cbind(Z,cset)
```

```
}
main_effects = paste(names(Z),collapse="+")
form1<-as.formula(paste(names(relfit),"~",main_effects,sep=""))
dset2<-as.data.frame(cbind(relfit,Z))
rsmod2<-lm(form1,dset2)
BAsum<-Anova(rsmod2, type="III")
# Pick off p-values from double regression
idx<-num_traits+2
BApval<-matrix(rep(0.0, num_traits), ncol=num_traits)
BAtstat<-matrix(rep(0.0, num_traits), ncol=num_traits)
for(i in 1:num_traits){
  for(j in i:num_traits){
    if(i==j){
      BApval[1,i]<-BAsum$"Pr(>F)"[idx]
      BAtstat[1,i]<-BAsum$"F value"[idx]
    }
    idx<-idx+1
  }
}
cat("Performing permutation test of canonical coefficients....May take awhile..be patient!\n")
main_effects = paste(names(strait),collapse="+")
form1<-as.formula(paste("permfit~",main_effects,sep=""))
temp_names<-c("Z_1")
numc<-ncol(y)
for(i in 2:numc){
  temp_names<-c(temp_names,paste("Z",i,sep="_"))
}
```

```
for (c in 1:num_perm){
 if((c%%piter)==0){
  cat("Iteration ",c," out of ",num_perm,"\n")
 }
 # Need to permute covariates along with fitness measure
 if(cov_flag==1){
  permset<-as.data.frame(cbind(relfit,cset))
  permset2<-permset[order(sample(permset[,1])),]
  permfit<-permset2[,1]
  end_pt<-ncol(permset2)
  cset_perm<-permset2[,2:end_pt]
  strait<-as.data.frame(cbind(jstrait,cset_perm))
  main_effects = paste(names(strait),collapse="+")
  form1<-as.formula(paste("permfit~",main_effects,sep=""))
 }else{
  permfit<-sample(relfit[,1])
 }

 # Fit RSM model to permuted dataset
 dset2<-as.data.frame(cbind(permfit,strait))
 rsmodp<-lm(form1,dset2)
 gamma<-matrix(rep(0.0, num_traits*num_traits), nrow=num_traits)
 num_terms<-ncol(strait)
 index<-num_traits+2
 for(i in 1:num_traits){
  for(j in i:num_traits){
   if(i==j){
```

```
      gamma[i,j]<-2.0*rsmodp$coefficients[index]
      gamma[j,i]<-gamma[i,j]
      index<-index+1
    }else{
      gamma[i,j]<-rsmodp$coefficients[index]
      gamma[j,i]<-gamma[i,j]
      index<-index+1
    }
  }
}
# Compute test statistics from double regression method
m<-eigen(gamma)$vectors
y<-as.matrix(strait[,(1:num_traits)])%*%m
for(i in 1:num_traits){
  for(j in i:num_traits){
    newterm<-y[,i]*y[,j]
    y<-cbind(y,newterm)
  }
}
Z<-as.data.frame(y)
names(Z)<-temp_names
if(cov_flag==1){
  Z<-cbind(Z,cset_perm)
}
main_eff = paste(names(Z),collapse="+")
form2<-as.formula(paste("permfit~",main_eff,sep=""))
dset2<-as.data.frame(cbind(permfit,Z))
```

```
  rsmod2<-lm(form2,dset2)
  BAsum<-Anova(rsmod2, type="III")
  # Pick off p-values from double regression
  idx<-num_traits+2
  p_tstat<-matrix(rep(0.0, num_traits), ncol=num_traits)
  for(i in 1:num_traits){
    for(j in i:num_traits){
      if(i==j){
        p_tstat[1,i]<-BAsum$"F value"[idx]
      }
      idx<-idx+1
    }
  }

  if(c==1){
    stat_track<-p_tstat
  }else{
    stat_track<-rbind(stat_track, p_tstat)
  }
}
cat("Finished with permutation testing....\n")
exceed<-matrix(rep(0.0,num_perm*num_traits), nrow=num_perm)
for(c in 1:num_perm){
  for(j in 1:num_traits){
    if(stat_track[c,j]>BAtstat[j]){
      exceed[c,j]<-1.0
    }
```

```
  }
}
pvalues<-matrix(rep(0.0, num_traits), ncol=num_traits)
for(c in 1:num_traits){
  pvalues[1,c]<-mean(exceed[,c])
}
results<-rbind(can_coef, pvalues)
rownames(results)<-c("Eigenvalues","Permutation p-values")
print(results)
cat("Eigenvectors of Gamma...\n")
eigvec<-as.data.frame(obs_m)
rownames(eigvec)<-names(traits)
print(eigvec)
```

APPENDIX B

Methods for sequential model building approach used to compare episodes of sexual selection – taken from Chenoweth and Blows 2005: -

We used a sequential model building approach to assess whether gradients of linear and non-linear sexual selection acting on male CHCs differed significantly between the two episodes of selection (Draper and John 1988; Chenoweth and Blows 2005). In short this sequential approach tests the difference in the sign and magnitude of the linear, quadratic and correlational selection gradients across the different episodes of selection by comparing the change in variance explained by a regression model that fits a single relationship through the two selection episodes being compared (model 1) to a regression model that fits a separate relationship for each episode of selection (model 2). If model 2 explains significantly more variance than model 1, as determined by a partial *F* test, this demonstrates that the selection gradients differ across selection episodes. We began by running a reduced regression model which included selection episode as a dummy variable (coded as ms or fs) and contained only the standardised linear terms:

$$S = \beta_0 + \alpha_0 Episode + \sum_{i=0}^{n} \beta_{i\,\mathrm{C}_i} + \varepsilon, \tag{1}$$

Where *S* was the binomial fighting/mating success measure, $\mathrm{C_i}$ refers to the log-contrast concentration of the *i*th principal component (PCs representing CHCs), *n* represented the number of PCs in the model and ε is the unexplained error. From (1), the unexplained (i.e. residual) sum of squares for this reduced model (SS_r) was compared to the same quantity (SS_c) from a second complete model that included all of the terms in (1) with the addition of the terms $\alpha_i C_i$Epsiode, which represents the linear interaction of the dummy variable, selection episode, and the *i*th PC:

$$S = \beta_0 + \alpha_0 Episode + \sum_{i=0}^{n} \beta_{i\,\mathrm{C}_i} + \sum_{i=0}^{n} \alpha_{i\,\mathrm{C}_i} Episode + \varepsilon, \tag{2}$$

A partial *F*-test (Bowerman and O'Connell 1990) was used to compare SS_r and SS_c from (1) and (2) respectively, to test whether linear sexual selection on male CHCs differed between the selection episodes:

$$F_{a,b} = \frac{(SS_r - SS_c)/a}{SS_c/b} \tag{3}$$

where *a* is the number of terms that differ between the reduced and complete model and *b* is the error degrees of freedom for SS_c.

To test whether the quadratic gradients of selection acting on male CHCs differed between selection episodes, the SS_r from the reduced model:

$$S = \beta_0 + \alpha_0 Episode + \sum_{i=0}^{n} \beta_{i\,C_i} + \sum_{i=0}^{n} \alpha_{i\,C_i} Episode + \sum_{i=0}^{n} \beta_i C_i^2 + \varepsilon, \tag{4}$$

was compared to the SS_c of the complete model:

$$S = \beta_0 + \alpha_0 Episode + \sum_{i=0}^{n} \beta_{i\,C_i} + \sum_{i=0}^{n} \alpha_{i\,C_i} Episode + \sum_{i=0}^{n} \beta_i C_i^2 + \sum_{i=0}^{n} \beta_i C_i^2\ Episode + \varepsilon, \tag{5}$$

using (3).

Finally to test whether correlational selection gradients different significantly between selection episodes, the SS_r from the reduced model:

$$S = \beta_0 + \alpha_0 Episode + \sum_{i=0}^{n} \beta_{i\,C_i} + \sum_{i=0}^{n} \alpha_{i\,C_i} Episode + \sum_{i=0}^{n} \beta_i C_i^2 + \sum_{i=0}^{n} \beta_i C_i^2\ Episode + \sum_{i=0}^{n} \sum_{j\geq 1}^{n} \beta_{ij} C_i C_j + \varepsilon, \tag{6}$$

was compared to the SS_c of the complete model,

$$S = \beta_0 + \alpha_0 Episode + \sum_{i=0}^{n} \beta_{i\,\mathrm{C}_i} + \sum_{i=0}^{n} \alpha_{i\,\mathrm{C}_i} Episode + \sum_{i=0}^{n} \beta_i\, \mathrm{C}_i^2 + \sum_{i=0}^{n} \beta_i\, \mathrm{C}_i^2 \; Episode + \sum_{i=0}^{n} \sum_{j\geq 1}^{n} \beta_{ij} C_i C_j + \sum_{i=0}^{n} \sum_{j\geq 1}^{n} \alpha_{ij} C_i C_j Episode + \varepsilon, \tag{7}$$

In summary, the comparison of model (1) versus (2), (4) versus (5), and (6) versus (7) provides a test for the overall significance of the interaction between selection episode and the linear, quadratic and correlational selection acting on male CHCS, respectively. Therefore significant differences in these model comparisons (as detected by a partial *F*-test) demonstrate that the linear, quadratic and/or correlational selection gradients imposed by the selection episodes differ, respectively. We also inspected the interaction of individual principal components with the selection episodes from the full model (7) to determine which of the PCs were responsible for the significance of the overall partial *F*-test.

APPENDIX C

CHC profile composition: -

Figure S1. A typical GC profile obtained from solvent extracts of the cuticle of male *Gnatocerus cornutus*. The *x*-axis shows the retention time (in minutes) and the *y*- axis shows the GC signal strength (in picoamperes). We found 24 unique CHC peaks in addition to the internal standard (pentadecane, peak not shown), which are characterised according to their mass spectra in Table S1.

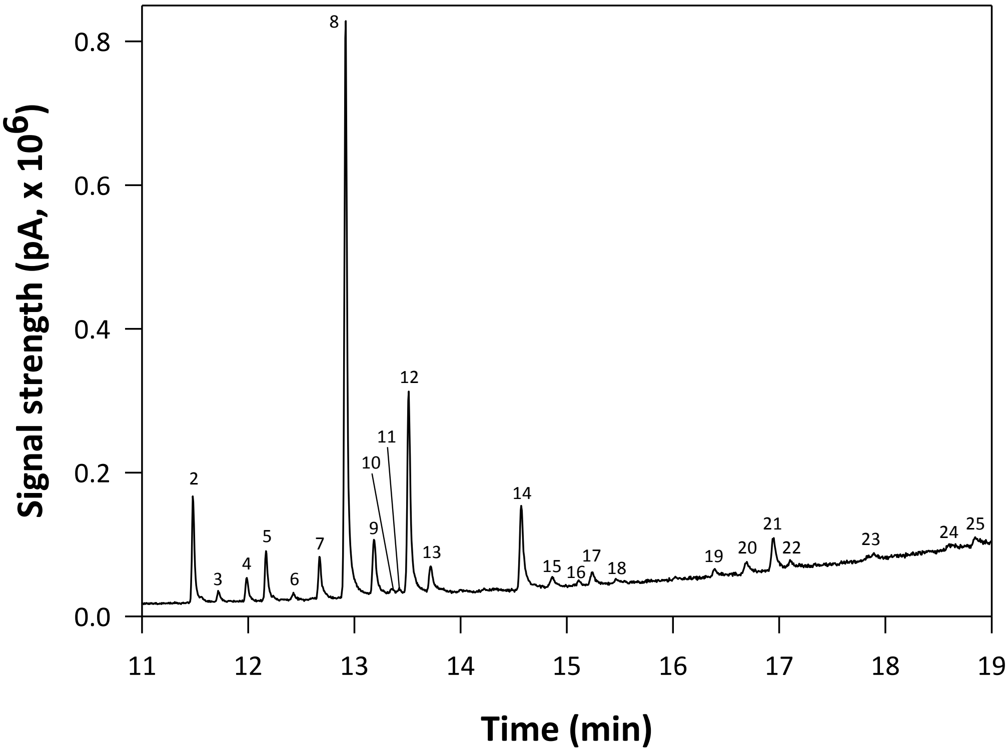

Table S1. Chemical characterization of male CHCs in *Gnatocerus cornutus*. KRI: Kovats Retention Index for each chemical compound, DMDS: diagnostic ions used for compound identification after derivation with dimethyl disulphide.

Peak	KRI	Compound	Diagnostic ions
1	2495	C_{25}	352
2	2531	11-MeC_{25}	168, 227, 351
3	2568	3-MeC_{25}	337, 57, 351
4	2594	C_{26}	366
5	2629	11-MeC_{26}	168, 238, 365
6	2661	5-C_{26}-ene	DMDS: 458, 117, 341
7	2693	C_{27}	380
8	2728	11-MeC_{27}	168, 252
9	2748	Unknown	
10	2759	11,15-diMeC_{27}	267, 168, 197, 239
11	2769	3-MeC_{27}	365, 57
12	2794	C_{28}	394
13	2894	C_{29}	408
14	2927	13-MeC_{29}	252, 196
15	2956	11,15-diMeC_{29}	295, 168, 224, 239
16	2969	3-MeC_{29}	57, 393
17	2993	C_{30}	422
18	3093	C_{31}	436
19	3126	15-MeC_{31}	224, 252
20	3152	3,19, 3,17-diMeC_{31}	196, 224, 267, 295, 435
21	3169	3-MeC_{31}	57, 421
22	3250	4,12-diMeC_{31}	435, 71, 309, 197
23	3325	11-MeC_{33}	169, 337, 225, 281
24	3349	15,17-diMeC_{33}	295, 225, 253, 267
